# The effect of action observation and motor imagery on jumping and perceived performance

**DOI:** 10.3389/fpsyg.2024.1362976

**Published:** 2024-07-09

**Authors:** Muhammet Cihat Çiftçi, Baki Yılmaz

**Affiliations:** Faculty of Sport Science, Department of Sports Management, Ankara Yıldırım Beyazıt University, Ankara, Türkiye

**Keywords:** action observation, exercise and sports psychology, jumping performance, motor imagery, motor skills

## Abstract

**Introduction:**

Action observation (AO) and motor imagery (MI) are cognitive processes that involve mentally rehearsing and simulating movements without physically performing them. However, the need for the evidence to support influence of imagery on performance is increasing. This study aims to investigate the impact of combining motor imagery with action observation on athletes’ performance and performance perception.

**Method:**

Using a pre-test post-test design with a factorial setup, participants were randomly assigned to experimental and control groups. A pre-research power analysis determined the sample size, resulting in 21 voluntary participants (10 male). Opto Jump device recorded drop jump performance measurements, while participants predicted their performance post-motor imagery and action observation practices. The experimental group underwent an 8-week AOMI intervention program, involving 24-minute motor imagery sessions during video observation thrice weekly. Post-test measurements were taken after the intervention.

**Results:**

Results indicated no significant performance increase in the experimental group post-intervention, yet the group showed enhanced performance estimation following the video observation, but not in motor imagery condition. Conversely, this improvement was absent in the control group.

**Discussion:**

Although AOMI intervention didn’t enhance physical performance, it has positively affected athletes’ perception toward their performance. The findings are discussed in relation to existing literature.

## Introduction

Motor simulation theory suggests that individuals can mentally rehearse an action both overtly and covertly through action observation (AO) and motor imagery (MI) in the absence of motor execution (Jeannerod, 1994, 2001). AO and MI describes the simultaneous act of generating, sustaining, and modifying a movement while following a kinesthetic representation of the same action synchronized in time (Vogt et al., 2013). Meers et al. (2020) introduced the visual guidance hypothesis (VGH) as an explanation for how AOMI may impact movement. They propose that during AOMI, motor imagery takes precedence, with the action observation component serving merely as an external visual guide that enhances the vividness of MI generation. This suggests that AO does not activate a separate motor representation during AOMI, but rather reinforces the motor representation stemming from MI. VGH posit that AOMI has the potential to influence motor skill execution beyond isolated AO or MI by enhancing activity in motor regions of the brain.

Studies evaluating the impact of the AOMI approach on various domains (neurological, behavioral, psychological) exist in the literature. When examined at from a cognitive perspective, motor learning is influenced by changes in mental representations of motor skills (Schack, 2004). These mental representations consist of cognitive information related to executing the movement, including required body postures, relevant movement components, and associated sensory outcomes (Wright et al., 2022). For instance, when a skill is about to be performed by an athlete, the movement is guided by evoking mental representations of the skill. Considering that AO and MI develop mental representations through different mechanisms, combining these two processes in an AOMI intervention is suggested to be more effective than independent AO or MI in enhancing the mental representations of the action in long-term memory (Kim et al., 2017; Wright et al., 2022). From neurological perspective, motor simulation theory explains how activations in the neural network system may cause changes. In the past decade, research on AOMI interventions has consistently shown increased activity in motor regions of the brain compared to independent AO or MI. The evidence suggests an activation in overlapping brain regions during the imagery, observation, or execution of movements (Hétu et al., 2013; Hardwick et al., 2018; Wright et al., 2018). However, the direct link between increased neurophysiological activity during AOMI and improved performance is yet to be established (Frank et al., 2020). However, the similarity in neural activation during imagery does not fully explain skill development in sports performance and sport-specific contexts. The processes of planning, programming, monitoring, and controlling movements during motor imagery suggest that the underlying mechanism of this method is much more diverse and intricate (see e.g., O’Shea and Moran, 2017). The discussion toward motor simulation theory raised by O’Shea and Moran (2017) highlights the importance carefully examining the causes of behavioral consequences of imagery interventions.

In terms of behavioral outcomes, Smith and Holmes (2004) demonstrated that participants receiving AOMI intervention showed higher performance in golf putting tasks compared to those engaged solely in motor imagery. Similarly, in a study by Wright and Smith (2009), participants receiving an intervention similar to AOMI exhibited increased forearm strength compared to those engaged only in motor imagery. The effectiveness of AOMI in enhancing performance stems from eliminating the necessity to create mental movement imagery through visual input (Holmes and Calmels, 2008). Hence, participants do not exert additional effort to generate mental images of the desired movement, allowing them to focus on the kinesthetic aspects of the desired skill by clearing attentional resources. Moreover, visual, auditory, or temporal cues related to motor skills can be conveyed to participants through video (Eaves et al., 2016b). Studies examining the acute effects of imagery interventions on performance generally provide evidence related to cognitive components in tasks such as balance, movement precision (Smith and Holmes, 2004; Romano-Smith et al., 2018; Wright et al., 2018), or predominantly maximal isometric muscle contractions (Wright and Smith, 2009; Di Rienzo et al., 2015, 2019). A detailed evidence can be found in the meta-analysis conducted by Chye et al., 2022 where authors suggest that AOMI influences sport-specific movement outcomes moderate to high-level. It can be drawn from the existing literature that the effectiveness of imagery interventions on jumping requires attention.

Jumping, a complex human movement that necessitates intricate motor coordination between upper and lower body segments, involves the assessment of the propulsive force of the lower extremities during vertical jumps to assess the explosiveness of both sedentary individuals and elite athletes (Markovic et al., 2004). In this study, jumping performance was chosen as a fundamental measurement tool due to its widespread use in evaluating sports performance and commonly being a parameter used to evaluate athletes’ physical abilities. Especially considering the importance of jumping ability in terms of many sports performances (Young et al., 1995; Gorostiaga et al., 2004; Walsh et al., 2004; Mikkola et al., 2007), examining motor imagery in this context is necessary as well. However, to the best of our knowledge, the number of studies examining the effect of imagery on jumping performance is quite limited in the sports psychology literature. The closest studies have usually examined the effect of motor imagery on jumping performance.

A recently conducted meta-analysis by Lindsay et al. (2023) included only one study (Olsson et al., 2008) examining the impact of imagery on jumping performance. In this study by Olsson et al. (2008), high jumpers showed no significant difference in jump heights following imagery sessions, yet the group practicing imagery in addition to training showed a notable improvement in technical skill, particularly in bar clearances. Another study by Battaglia et al. (2014) with gymnasts revealed a positive impact of motor imagery on jumping performance parameters. On the contrary, Avila et al. (2015) found no significant difference in active jumping performance between groups practicing imagery compared to those who did not. Additionally, Bergmann et al. (2013) examined the effect of motor imagery on depth jumps, indicating a rather limited effect. They propose that in addition to, participants’ predisposition toward the motor skill used during the experimental task might have influenced this outcome.

The utilization of the jumping paradigm in this research aligns with the strength approach to assess the effectiveness of AOMI intervention. This choice is deliberate because prior imagery research predominantly employed tasks with a substantial cognitive aspect, such as balance, precise movements, or maximal isometric muscle contractions (Bergmann et al., 2013; Hardwick et al., 2018; Di Rienzo et al., 2019; Simonsmeier et al., 2021). AOMI could be an important tool for enhancing jumping performance because the nature of the jumping movement requires the interaction of both visual and kinesthetic elements (Wright et al., 2022). AOMI can support the formation of mental representations of the jumping movement by observing the correct form and technique of the jump (Eaves et al., 2016a; Kim et al., 2017; Bach et al., 2022). This method can enhance movement performance by increasing brain activation of the muscle groups actively used during jumping. AOMI can help automate motor skills and enhance performance by mentally rehearsing the movement (Nedelko et al., 2012; Lee et al., 2021). AOMI can provide athletes with visual feedback to understand and optimize the complexity of the jumping movement (Kraeutner et al., 2020).

Although many studies suggest the impact of AOMI interventions on performance (Eaves et al., 2016b; Simonsmeier et al., 2021; Chye et al., 2022), varying outcomes regarding its influence specifically on jumping performance indicate a lack of consensus (Olsson et al., 2008; Bergmann et al., 2013; Battaglia et al., 2014; Avila et al., 2015). Despite the strong evidence supporting the AOMI effectiveness in certain movement classifications the lack of consensus in jumping performance literature requires more attention. Based on this, we developed a paradigm to assess the perception of the participants toward the intervention. This discriminate our experiment in terms of finding the perceived benefit of the AOMI interventions from individual perspective. By doing so, we hypothesized that individuals in intervention group would perceive the imagery more beneficial compared to their counterparts. It is also essential to note that measuring a motor skill which is widely accepted as an indicator of athletic performance is a necessity of imagery studies to highlight its’ effectiveness. Therefore, we tend to believe that conducting research involving imagery intervention utilizing the Action Observation with Motor Imagery (AOMI) method may influence performance. Therefore, the need arose to conduct an eight-week intervention study incorporating AOMI intervention to investigate its potential impact on performance.

## Materials and methods

### Research design

A randomized controlled 2 × 2 factorial design was used in this experimental study (Goodwin and Goodwin, 2016; Passer, 2020). The independent variables in this study were (1) type of intervention (AOMI vs. control) and (2) time (pre-test vs. post-test). The dependent variables were (1) drop jump performance, as measured by the Opto Jump device, and (2) perceived performance estimation, as predicted by the participants after the intervention. This design allowed us to investigate both the main effects of each independent variable and the interaction effects between them on the dependent variables. By employing this factorial design, we were able to systematically explore the influence of the combined AOMI intervention on both objective performance metrics and subjective performance perceptions.

### Participants

Students from the faculty of sport sciences were recruited for the study. Inclusion criteria stipulated participants to be actively involved in sports, right-handed, free from neurological conditions, and possessing normal visual acuity. *A priori* power analysis determined that a group size of 20 participants was adequate (Effect size *f* = 0.35, α = 0.05, Power = 0.80). 30 participants (15 males) were hired for study to account for possible dropouts. Eight participants dropped out of the study and one outlier from the experimental group was excluded based on statistical methods leaving 21 participants (10 males, 11 females) for final analysis. The experimental group had an average age of 20.20 years (SD = 1.81), height of 171.30 cm (SD = 6.44), and weight of 62.70 kg (SD = 13.89). The control group had an average age of 19.09 years (SD = 0.53), height of 178.18 cm (SD = 7.76), and weight of 72.27 kg (SD = 6.88). Imagery ability, assessed using the MIQ-R, showed pre-test kinesthetic scores of 5.95 (SD = 0.80) and post-test scores of 6.00 (SD = 0.62) for the experimental group, compared to 5.43 (SD = 1.01) and 5.55 (SD = 0.52) for the control group. Pre-test visual scores were 6.42 (SD = 0.44) and post-test scores were 6.20 (SD = 1.40) for the experimental group, while the control group had pre-test scores of 6.30 (SD = 0.56) and post-test scores of 6.30 (SD = 0.70).

#### Measurements

##### Movement imagery questionnaire-revised form

Participants’ motor imagery abilities were assessed using the Movement Imagery Questionnaire-Revised Form (MIQ-R), adapted into Turkish by Akkarpat (2014) from Monsma et al. (2009)’s original questionnaire. This tool evaluates visual and kinesthetic imagery across four movements: jumping, arm movement, toe touching, and knee raising. Participants completed three stages for each movement: assuming the initial position, physically performing the movement, and mentally recreating the movement without executing it physically. They rated the ease of visualizing/feeling each movement on a scale from 1 to 7. To ensure understanding, participants were given a demonstration and allowed to physically perform the movements before the assessment.

##### Likert measurements

Participants were requested to provide self-assessments of their motivation levels throughout the course of the experimental procedures. To facilitate this evaluation, participants were instructed to employ a 10-point Likert scale, with ratings ranging from 1 (“indicating a low level of motivation”) to 10 (“indicating a high level of motivation”). Additionally, participants were asked to evaluate the mental difficulty they perceived during each of the imagery sessions. This assessment was conducted using a single statement: “What is the perceived level of mental difficulty experienced during the imagery activity you engaged in?” Responses were recorded on a 10-point Likert scale, spanning from 1 (“indicating not at all difficult”) to 10 (“indicating a very high level of difficulty”). It’s noteworthy that this methodology, involving a single question to assess motivation in relation to experimental processes and perceived difficulty during imagery sessions, has been previously utilized in an imagery study conducted by Di Rienzo et al. (2019).

##### Performance measurement

Participants underwent three weeks of practice trials for Drop Jump (DJ) before the pre-test measurements. Both groups familiarized themselves with these jumps and performed them in consistent environmental conditions. Before the jumps, participants completed warm-up exercises, including stretches, body-weight squats, and a short run. The DJ measurements followed a full rest period after the warm-up, using the OptoJump Next^®^ device. This device has high reliability (ICC 0.88–0.98), validated in sports sciences (Glatthorn et al., 2011; İnce, 2019) and was used with manufacturer-advised protocols for DJ measurements. During assessments, participants executed a vertical jump at maximum speed, adhering to specific body positions. Any deviation led to exclusion or repeat of measurements, with the average of two successful trials determining performance (Battaglia et al., 2014).

### Perceived performance measurement

Following the completion of physical performance measurements, perceived performance predictions were measured. During both the pre-test and post-test stages, each participant was instructed to indicate their perceived performance with implementing single bout of action observation and motor imagery practice. While observing the video participants were tried to imagine drop jump and indicate how high they might have jumped in Action Observation Perceived Performance (AOPP) condition. During the AOPP session, participants were asked to conduct simultaneous action observation and motor imagery. As for the Motor Imagery Perceived Performance (MIPP) condition, they executed a single bout of imagery of without visual aid. In the MIPP condition, participants were first instructed to mentally rehearse the jumping task and then indicate their perceived jump scores. Each participant completed single bout of AOP and MIP trial in random order during both the pre-test and post-test measurement sessions to prevent sequential effects. Perceived performance measurement wasn’t repeated throughout the intervention phase. This method ensured consistent measurement of perceived performance in both stages of the study.

### Action observation and motor imagery intervention

After the pre-test measurements, the experimental group commenced the intervention program with meticulously produced videos displaying DJ movement, featuring selected models from the Faculty of Sports Sciences to match participants’ physical abilities. Male models were used for male participants and female models for females, enhancing behavioral congruence. These movements were recorded at 60 frames per second using a GoPro Hero 5 Black action camera. In alignment with established literature recommendations, the videos were recorded from a first-person perspective due to its known efficacy in more profoundly engaging the motor system (Alaerts et al., 2009). This perspective not only provides a closer behavioral match but also significantly enhances overall efficacy (Wakefield et al., 2013).

Before the intervention, participants received comprehensive training in imagery techniques, including the PETTLEP model, emphasizing sensory effects like environmental stimuli, muscle activation, heart rate, and postural changes (Lang et al., 1992; Romano-Smith et al., 2018). Prioritizing sensory effects significantly improves imagery and animation skills (Williams et al., 2013) and animation skills (Wakefield and Smith, 2009).

The intervention ran three times weekly for 8 weeks, following prior research recommendations (Toth et al., 2020; Lee et al., 2021). Each 24-min imagery session comprised 4-min segments with 1-min breaks (4 min. 6 blocks totalling 30 min. including the breaks), based on guidance from Lee et al. (2021). Sessions were held in the same laboratory as the initial measurements. The practitioner overseeing the sessions was blind to specific research objectives, aiding participants through each 4-min block and emphasizing the importance of the 1-min breaks.

### Manipulation check

Imagery diaries served as a manipulation control in the study. Participants were provided with diaries and instructed to complete them to verify adherence to the imagery session instructions throughout the study’s duration. Furthermore, it is worth noting that maintaining diaries to document specific emotional experiences during each imagery session, any encountered difficulties, or feedback on the intervention method, aligns with practices in prior imagery research (Smith et al., 2020).

#### Procedure

The participants, upon giving their consent, were enrolled in the study and assigned to either the experimental or control group using the last four digits of their student numbers. They were informed about the withdrawal option and confidentiality measures. Participants attended the laboratory for eight weeks to follow the intervention program. The study’s nature was kept confidential until data collection was complete, focusing on investigating imagery’s impact on psychological variables. Initial assessments involved imagery ability measurement with the Movement Imagery Questionnaire-Revised Form. Participants were familiarized with performance measurements using the Opto Jump device for three weeks. DJ exercises were practiced 3 times for each week during familiarization. Pre-test measurements were taken after a 7-day rest period to avoid muscle strain. Practice sessions aimed to mitigate bias from physical predispositions, learning effects, and performance variations. Following familiarization, pre-test measurements were implemented along with the perceived performance prediction estimations. They were welcomed by the first author when they arrived the laboratory and informed about the test procedures. Participants underwent 10 min warm-up sessions before engaging in drop jumps. They were considered to be ready to perform the tests after having a short rest (3–5 min.) from warm-up. The AOMI intervention was initiated following the completion of pre-test measurements. Participants were instructed to practice action observation and motor imagery during intervention sessions with fully focus on the movement. Sessions were supervised in a distraction-free laboratory setting. The PETTLEP principles were introduced and ensured to be implemented, assisting participants in adapting these principles to their imagery skills.

In the pre and post-test participants tried to predict their jump heights after a single bout of AOP and MIP in random order. In the AOP performance estimation attempt, they observed a video of model athlete performing the task. First author, who also conducted the tests, provided feedback on jump height and ground contact time durations but this feedback was not provided in the MIP attempts. During the intervention imagery sessions lasted 24 min, divided into 4-min blocks with 1-min breaks in Kraeutner et al. (2020). At each session’s end, a verbal imagery scenario was presented as a warm-up exercise, followed by watching jump videos, simulating drop jumps in sync with the displayed video. Completion of a 30-min session equated to one Action Observation and Motor Imagery (AOMI) intervention session. At the end of the study, participants were thanked and debriefed about the experimental procedure (Supplementary Table 1).

**TABLE 1:** Comparison of participants’ demographic variables.

Group	Experimental		Control		Independent sample *t*-test	
**Variables**	**Mean**	**Sd.**	**Mean**	**Sd.**	***t***	***p***
Age (years)	20.20	1.81	19.09	0.53	1.861	0.091
Height (cm)	171.30	6.44	178.18	7.76	−1.722	0.101
Weight (kg)	62.70	13.89	72.27	6.88	−1.970	0.071
Number of exercises per week	4.10	1.10	4.45	0.68	−1.308	0.216
Training duration per session	62.50	15.85	68.63	13.24	−0.966	0.346
Pre-test MIQ-R (kinesthetic)	5.95	0.80	5.4318	1.01	1.283	0.215
Post-Test MIQ-R (kinesthetic)	6.00	0.62	5.5455	0.52	1.817	0.085
Pretest MIQ-R (visual)	6.42	0.44	6.2955	0.56	0.579	0.569
Post-Test MIQ-R (visual)	6.20	1.40	6.2955	0.70	−0.199	0.844
Motivation to research participation	8.90	1.10	8.455	1.75	0.689	0.499

### Statistical analysis

SPSS version 22 was used for data analysis, presenting descriptive statistics (means and standard deviations). Normality was confirmed in dependent variables for both groups using the Shapiro–Wilk test, histograms, and probability plots. We identified the extreme outlier statistically by assessing histograms, probability plots, and conducting the Shapiro–Wilk test for normality, confirming its status based on 1.5 times the interquartile range (IQR) thresholds, and subsequently excluded it from the dataset. For detecting possible differences between groups, independent samples *t*-tests were employed at pre-test. The two-way mixed ANOVA (2 × 2) was implemented to find intervention effect and between-group differences.

We utilized the Intraclass Correlation Coefficients (ICC) to assess test-retest reliability, following Koo and Li’s (2016) classification system. Based on their work, ICC values were categorized as follows: ≤ 0.49 poor, ≥ 0.50 ICC < 0.75 moderate, ≥ 0.75 ICC < 0.9 (good), and ≥ 0.9 excellent (Koo and Li, 2016). Cohen’s d effect sizes were used to determine the impact of the independent variable on dependent variables between pre-test and post-test measurements. These effect sizes were accompanied by their 95% confidence intervals. Cohen’s classifications—small (0.2), medium (0.5), and large (0.8)—were adopted to interpret the effect sizes (Cohen, 2013). A significance level of *p* < 0.05 was used for all statistical tests.

## Results

### Normality tests

The Shapiro–Wilk test confirmed normal distribution for most pre-test and post-test data, validating the use of parametric tests. Deviation in one parameter’s pre-test values resulted from a single outlier but didn’t significantly affect overall normality. Hence, outlier removal was deemed unnecessary. Participant demographics indicated diverse sports backgrounds in both groups. Exercise frequency for the experimental group was 3.70 ± 1.70 times/week (62.50 ± 15.85 mins/session) and for the control group, 4.45 ± 0.68 times/week (68.63 ± 13.24 mins/session). No significant difference in motivation levels between groups was observed (*t* = 0.689, *p* = 0.499). Participants displayed consistent kinesthetic and visual imagery abilities across pre-test and post-test phases (kinesthetic: *t* = 1.28, *p* = 0.215, *t* = 1.817, *p* = 0.085; visual: *t* = 0.579, *p* = 0.569), indicating stable imagery skills. Additional demographic details are provided in Table 1.

### Intra-class correlation coefficient

The intraclass correlation coefficient (ICC) was computed to assess the reliability of the measurements. The ICC value obtained for jump performance was calculated as reliable scores (ICC ≥ 0.88) except for the DJ GCT. This high ICC values signifies that the measurements are consistent and reliable in actual performance recordings. Conversely, ICC obtained for performance predictions showed poor reliability (ICC ≤ 0.49) (Table 2).

**TABLE 2:** Demonstration of intra-class correlation coefficients.

Variable		ICC	95% confidence interval		F test with true value 0			
			Lower bound	Upper bound	Value	df1	df2	*p*
DJ MI PPP	Single measures	0.197	−0.201	0.558	1.537	20	20	0.172
DJ AO PPP	Single measures	0.496	0.084	0.761	2.888	20	20	0.011
DJ ELV	Single measures	0.880	0.762	0.946	40.487	20	60	0.000
DJ FT	Single measures	0.890	0.777	0.951	44.791	20	60	0.000
DJ GCT	Single measures	0.681	0.496	0.834	9.548	20	60	0.000

### Performance results

The repeated measures multivariate analysis of variance (ANOVA) conducted to assess the impact of the AOMI intervention program on performance revealed a significant main effect of time on drop jump performance in within-group comparisons [*F*(1, 19) = 14.491, *p* = 0.001, ηp2 = 0.433]. Conversely, there was no significant main effect found between groups [*F*(1, 19) = 0.885, *p* = 0.359, ηp2 = 0.045]. However, significant main effect was found in the group-time interaction [*F*(1, 19) = 4.435, *p* = 0.049, ηp2 = 0.189]. Pairwise comparisons were conducted to assess differences between experimental and control groups at two time points for the main interaction effect (Supplementary Figure 1). The results indicate no significant differences between groups at either time point (Pre-test: MD = −3.017, SE = 2.414, *p* = 0.227, 95% CI [−8.069, 2.035]; Post-test: MD = −1.184, SE = 2.126, *p* = 0.584, 95% CI [−5.634, 3.266]). All comparisons were adjusted using Bonferroni correction for multiple comparisons.

Supplementary Figure 2 demonstrates that there was no significant main effect of time in the within-group comparisons for the DJ AO perceived performance prediction [*F*(1, 19) = 0.085, *p* = 0.774, ηp2 = 0.004]. Similarly, no significant main effect was found between the groups [*F*(1, 19) = 0.141, *p* = 0.711, ηp2 = 0.007]. However, upon examining the group-time interaction, a significant interaction effect was found [*F*(1, 19) = 5.795, *p* = 0.026, ηp2 = 0.234]. The mean of the experimental group was significantly increased from pre-test (M = 28.1, sd = 2.33) to post-test (M = 30.95, sd = 7.37) compared to control group. There was no significant main effect of time within-group comparisons for the DJ MI perceived performance prediction [*F*(1, 19) = 2.791, *p* = 0.111, ηp2 = 0.128] and no significant main effect between groups was found [*F*(1, 19) = 0.705, *p* = 0.411, ηp2 = 0.036] (Supplementary Figure 3). Group-time interaction was also not significant in the same variable [*F*(1, 19) = 0.003, *p* = 0.955, ηp2 = 0.001].

## Discussion

This study investigates the impact of motor imagery during movement observation on jump performance over a 12-week period. The main outcome of the study points out that experimental group receiving AOMI intervention experienced lower decline in jump performance from pre to post-test compared to the control group but the difference was not significant. This finding indicates that, contrary to our hypothesis, the AOMI intervention did not enhance drop jump performance as expected. Given these results, further investigation is needed to understand the underlying mechanisms and potential factors influencing the effectiveness of AOMI interventions on athletic performance. The findings of our study seems to be contradictory to several previous studies (Yue and Cole, 1992; Ranganathan et al., 2004; Collet et al., 2011; Di Rienzo et al., 2015, 2019; Iacono et al., 2021). A study by Smith et al. (2020) suggested an increase in biceps strength following imagery intervention. Although various studies support the opposite, a meta-analysis conducted by Paulo Manochio et al. (2015) suggests that the evidence does not support the idea of imagery being an effective tool to enhance strength gains. Inconsistent results in the literature raise the question of whether imagery interventions, whether they include video observations or not, indirectly facilitated improvements in participants’ motor skills. However, due to the absence of video analysis methods, technical alterations related to jump performance could not be determined. For instance, Olsson et al. (2008) observed advancements in athletes’ technique despite no increase in jump heights in their study. Although our study’s results align with the absence of an increase in jump heights, the inability of our study to detect sport-specific technical improvements differs. This suggests that while the AOMI intervention may not affect performance directly, it may contribute to learning outcomes in jumping technique. Nonetheless, we did not measure biomechanics in our study to support this notion. In general, the data imply that the sole implementation of AOMI for imagery did not necessarily facilitate performance increase. These findings, consistent with certain studies (Smith et al., 2020), suggest limited performance changes when comparing imagery-only to physical application (Kraeutner et al., 2020).

Moreover, differences in the imagery protocols used, particularly in the content of instructions and mechanical characteristics of exercises, could influence the contradictory outcomes. Although the AOMI protocol outlined detailed movement steps, the absence of prior experience might have affected the performance. Concrete embodiment theories emphasizing the influence of prior experiences on learning processes (Mulder et al., 2004; Olsson and Nyberg, 2010; O’Shea and Moran, 2017; Iacono et al., 2021) and studies demonstrating how imagery builds upon previous physical experiences by modulating brain activation (Kraeutner et al., 2018). In another study by Collet et al. (2011), MI intervention was found to increase the number of maximum repetitions in leg press; however, no significant difference was observed in bench press between groups. Individual factors such as participants’ experience levels in resistance exercises and muscular adaptations might explain this discrepancy. These arguments contribute to our understanding of how previous physical experiences influence imagery and learning processes.

The question of how specific prior experience needs to be to influence imagery arises regarding its effectiveness. Olsson et al. (2008) compared high jump athletes with a control group of novices in an attempt to answer whether effectively imagining a skill previously experienced is feasible. Their study provided evidence that for the mental imagery of a complex skill, individuals need well-established motor representations, which subsequently transform into brain activities shaping motor representations. The findings suggested that imagery training reduced activity in the parietal cortex, resulting in more automatic imagery and a more easily accessible, efficient motor representation during motor performance. Considering the similar effects of observing and imagining movements, the role of physical experience during observation should also be considered. In a study by Aglioti et al. (2008), elite basketball players were compared with expert spectators (former basketball players who had stopped playing) and novices in predicting movement outcomes. The findings indicated that only elite basketball players were successful in predicting outcomes, suggesting that motor representations shaped by specific physical experiences are highly specific. Therefore, merely having prior high-level expertise in a particular task might not suffice for predicting the outcomes of movements; rather, the physical experience needs to be relatively recent. Balser et al. (2014) examined the impact of expertise on brain activation in expert volleyball and tennis players while predicting serve shots in both sports. Results showed that while athletes predicted serves faster in their own sport, neural activation in the observation network was higher in the unfamiliar sport. Additionally, Del Percio et al. (2008) highlighted that experienced athletes exhibited more effective neural cortical activity compared to novices, indicating higher neural focus. Hence, considering the relatively limited prior experience in active and depth jumps among our study participants and the time gap due to the pandemic before the research process, it is presumed that the lack of recent experience might have influenced our study’s outcomes.

In the study, participants were also asked to indicate their perception toward performance during imagery. They were tasked to estimate their performances after two different conditions: engaging in imagery and watching videos. Findings revealed that while participants predicted decreased jump scores, their estimated performance perceptions were higher in AO condition. This outcome is noteworthy, although the results was statistically insignificant. Participants reported a decline in imagery condition over time but noted an improvement in their performance after observing the role model. This indicates that relying solely on imagery might not foster performance necessarily, possibly due to participants struggling initially to clearly follow imagery instructions and control mental images (Holmes and Calmels, 2008). Additionally, there is a recognized limitation in researchers’ ability to monitor participants’ motor images (Wright et al., 2022). However, action observation might facilitate participants in better understanding and controlling their imagery performance (Olsson and Nyberg, 2010). Observation through video feedback enhanced participants’ performance. This suggests that conveying visual information allows participants to associate their kinesthetic sensations with the execution of the movement (Eaves et al., 2016a,b). As video observation is considered a passive process accessing the motor system through the observation of motor actions instead of imagery, this notion aligns with the mirror neuron system (Rizzolatti and Craighero, 2010).

The findings indicate the positive impact of video watching on the effectiveness of imagery in performance. However, uncertainties persist regarding whether subjective awareness serves as a precise mechanism to determine the completion of a movement simulation (O’Shea and Moran, 2017). The performance enhancement observed in video watching following the AOMI intervention provides significant hints on how visual inputs facilitate the improvement of participants’ mental representations and subsequently enhance their performance. Participants’ inclination to improve their performance by utilizing cognitive mental representations rather than directly controlling their movements warrants attention. Research emphasizes the activation of different mechanisms through visual and mental exercises (Kim et al., 2017). For instance, it is proposed that visual inputs could develop mental representations to sequence and time the components of movement execution (Frank et al., 2020). In this context, it is considered that visual inputs offer an advantage to the experimental group in creating relevant motor representations.

Additionally, individuals are reported to observe their own practices to enhance their performance and refine their visual perceptions (Hars and Calmels, 2007). Particularly, the perception of observing others as a means to enhance performance suggests an effective strategy for developing participants’ cognitive abilities. Participants who received AOMI intervention stated an improvement in their performance post-observation rather than during mental imagery, possibly indicating a more effective engagement of cognitive functions during simulation (O’Shea and Moran, 2017). Research suggests that observing movement activates different neural connections compared to motor imagery (Hardwick et al., 2018). According to the motor simulation theory, individuals can mentally construct images related to any motor skill, implying that visual inputs support learning (Jeannerod, 2001). This aligns with MST’s proposition that the restriction of implicit skills (execution of movement) during imagery is a part of the simulation process (Jeannerod, 2004, 2006).

Participants receiving AOMI intervention might have aimed to avoid engaging in the actual execution of movements during imagery rather than seeking performance enhancement. The constriction of movement during MI might reduce the possibility of carrying out the action by imposing limitations on cognitive processing. These constrictive processes are suggested to play a role in guiding motor orientation and inhibiting the execution of motor programs (Richard Ridderinkhof et al., 2011). Bach et al. (2014) discovered that participants found it more challenging to respond to stimuli with the limbs engaged in MI, leading them to respond with other body parts. This supports the close relationship between intentional processes in motor planning and constraining processes, potentially clarifying the results of the study. Furthermore, participants who received AOMI intervention expressing an increase in their performance post-observation might indicate the impact of video observation on their self-efficacy beliefs (Ste-Marie et al., 2012). Observation-based learning is highlighted to influence movement dynamics more than the output of movement, suggesting that those in the AOMI group might have perceived an improvement in their performance based on the role model in the video (Ashford et al., 2006).

### Limitations and implications

There are several implications and limitations that should be considered when reading this article. The lack of performance improvement and, in some cases, a decline observed in participants undergoing the 8-week AOMI intervention calls for careful consideration of these results. Anticipating that imagery alone would impact performance might lead to undesirable outcomes. The research also highlighted that AOMI intervention not only influenced performance but also affected perceptions toward performance. While experimental group (EG) participants predicted increased performance after video observation, the control group’s predictions were opposite. This indicates that AOMI over time positively influenced participants’ perceived benefit from the intervention. These outcomes suggest that in sports psychology practices, imagery interventions can be utilized as a tool to influence athletes’ self-efficacy perceptions.

Nonetheless, the study faced limitations that need to be considered in result interpretation. Demographic variations among participants could yield different results, particularly when applied to elite athletes or patient groups. Additionally, the absence of any physical training alongside the AOMI intervention restricts the evaluation to purely imagery-based effects. This study encountered constraints due to restricted access to long-term training routines for participants as there was last remaining effects of pandemic habits. This circumstance should be considered when interpreting results. Furthermore, the low intra-class correlation values imply the need for cautious evaluation of self-report performance predictions. The research highlighted that not all motor imagery programs utilized in practice exert the same level of influence on enhancing performance parameters. However, the specific reasons behind the varying efficacy levels among these programs weren’t extensively explored. Further investigation into the specific elements contributing to program effectiveness could provide a deeper understanding. The findings of the study emphasize the necessity to evaluate AOMI interventions across a broad spectrum of athletes to understand the effectives of it in difference disciplines.

The findings of this study indicate that motor imagery programs may not necessarily show a significant effectiveness in improving performance outcomes. It has been revealed that not every motor imagery program used in practice is equally effective in enhancing performance parameters. Therefore, decisions to choose motor imagery interventions for developing the performance should be based on after careful planning. Additionally, the study suggests that implementing motor imagery along with video observation may have positive effects on shifting participants’ perceptions of their performances. However, it is emphasized that when developing imagery intervention programs, applied psychologists must pay attention to participants’ prior experiences of relevant motor skills. The findings above shows important factors to consider when evaluating the effects of motor imagery programs on performance.

## Data availability statement

The raw data supporting the conclusions of this article will be made available by the authors, without undue reservation.

## Ethics statement

The studies involving humans were approved by the Social and Humanities Ethics Committee—Ankara Yıldırım Beyazıt University (Folder no: 2021-142, date: 15.03.2021-72). The studies were conducted in accordance with the local legislation and institutional requirements. The participants provided their written informed consent to participate in this study.

## Author contributions

MÇ: Conceptualization, Data curation, Formal analysis, Funding acquisition, Investigation, Methodology, Project administration, Resources, Software, Supervision, Validation, Visualization, Writing – original draft, Writing – review & editing. BY: Conceptualization, Investigation, Visualization, Writing – review & editing.
