# Supplementary material for: The effect of action observation and motor imagery on jumping and perceived performance
Source: Front Psychol. 2024 Jul 9;15:1362976. doi: 10.3389/fpsyg.2024.1362976 (PMC11263293; doi:10.3389/fpsyg.2024.1362976)
Supplement: Supplementary file 1 [file Data_Sheet_1.docx]

***Supplementary Material***

**Supplementary Figures and Tables**

**Table 1.**Diagrammatic representation of the experimental procedure period

| Group | Familiraztion Period | | | Pre-Test Measurement | 8 Week Intervention | Post Test Measurement |
| --- | --- | --- | --- | --- | --- | --- |
| Experimental Group | Week I | Week II | Week III | MIQ-R&  Performance Measurements of DJ and  Performance Predictions | AOMI Intervention of Drop Jump | MIQ-R&Performance Measurements of DJ and  Performance Predictions |
| Control Group |  |  |  |  | No Intervention |  |
