# Supplementary material for: The effect of action observation and motor imagery on jumping and perceived performance
Source: Front Psychol. 2024 Jul 9;15:1362976. doi: 10.3389/fpsyg.2024.1362976 (PMC11263293; doi:10.3389/fpsyg.2024.1362976)
Supplement: Supplementary file 2 [file Data_Sheet_2.docx]

Supplementary Material

**Supplementary Figures and Tables**


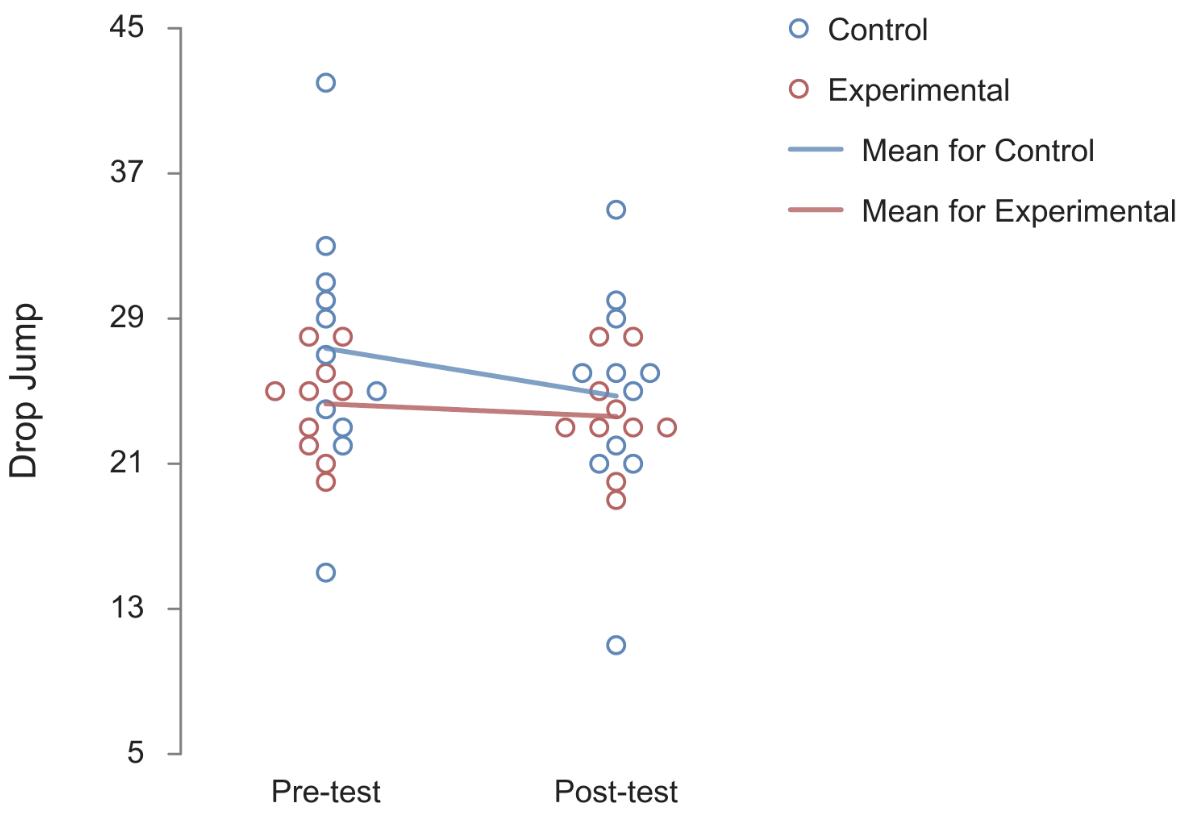


**Figure 1.** Comparison of pre-test and post-test heights in drop jump performance. Error bars represent standard deviations


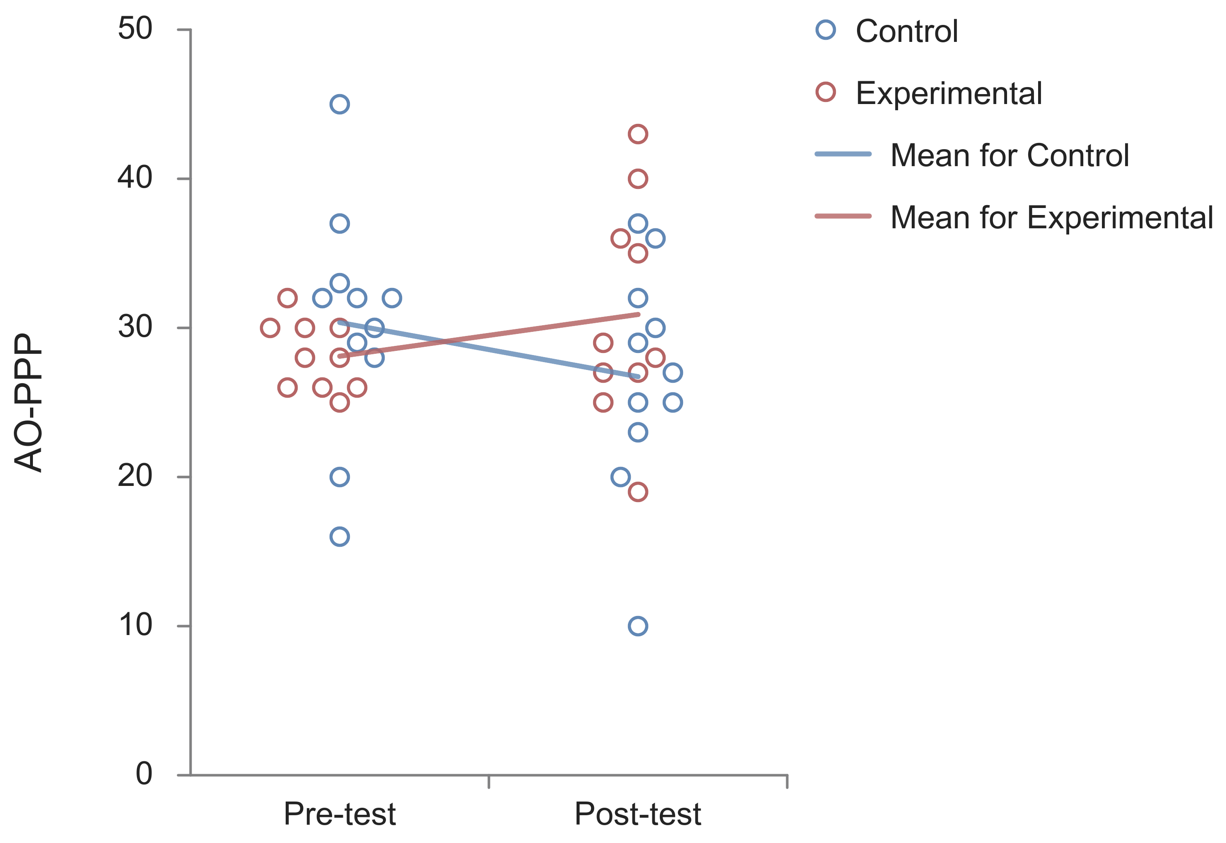


**Figure 2.** Demonstration of DJ AO perceived performance predictions of participants based on self-report evaluations.


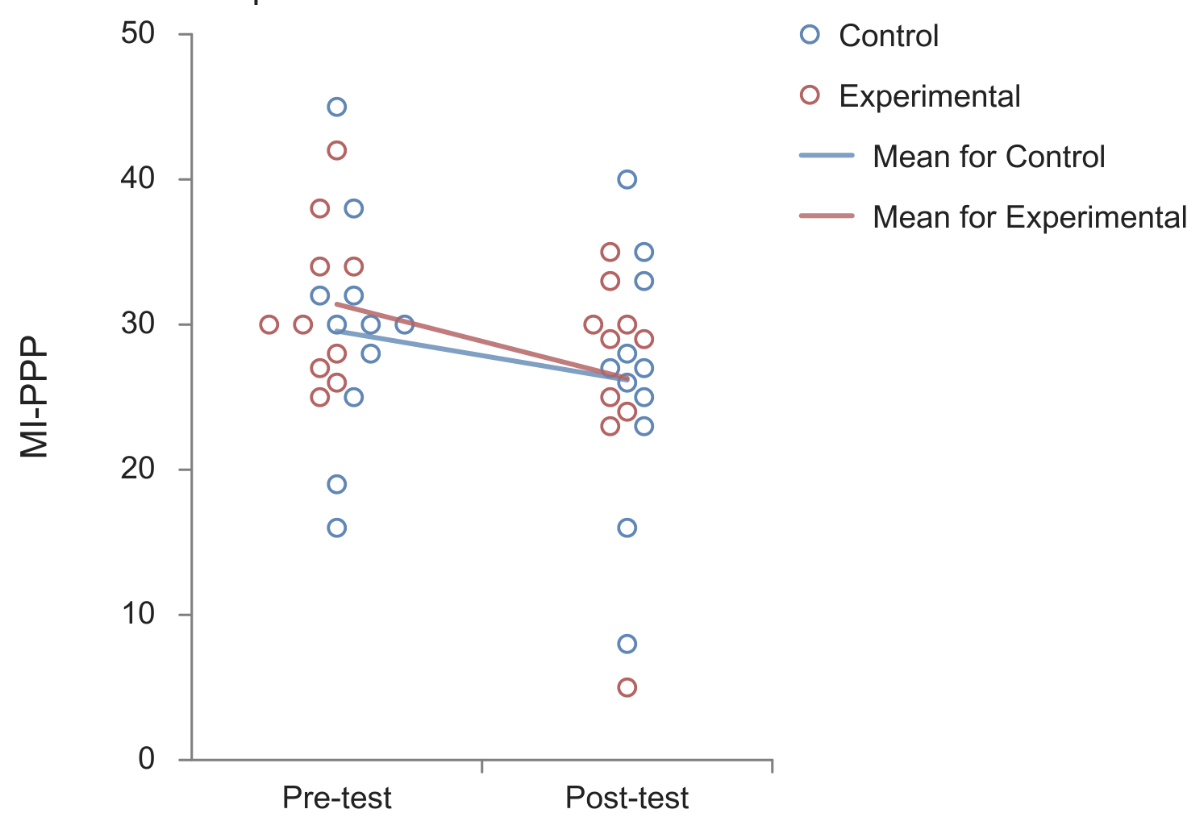


**Figure 3.** Demonstration of DJ MI perceived performance predictions of participants based on self-report evaluations.
